# Supplementary material for: Chitosan-Functionalized Poly(β-Amino Ester) Hybrid System for Gene Delivery in Vaginal Mucosal Epithelial Cells
Source: Pharmaceutics. 2024 Jan 22;16(1):154. doi: 10.3390/pharmaceutics16010154 (PMC10818660; doi:10.3390/pharmaceutics16010154)
Supplement: Supplementary file 1 [file pharmaceutics-16-00154-s001.zip › pharmaceutics-2780108-supplementary.pdf]

Supplementary materials

1. Synthesis of quaternized chitosan (QCS)

The original chitosan (20 g) was dissolved in up water containing 1% H_2O_2 (v/v) and stirred at 50 °C for 1 h, then cooled to room temperature and centrifuged to discard the supernatant. The precipitate was washed with deionized water, dissolved in deionized water containing 2% acetic acid (v/v). Then adjust the pH of the solution to 6.8 with NaOH, remove the precipitate, and continue to adjust to 8.0 to produce a large amount of precipitate. The precipitate was centrifuged and washed with deionized water and acetone to obtain degraded chitosan (CS) to improve solubility. CS (5 g, 0.0274 mol) was dissolved in 200 mL of 1% acetic acid aqueous solution (v/v), and then GTMAC (14.73 mL, 0.1098 mol) was added dropwise. After reacting at 55 °C for 20 h, the reaction solution was filtered and precipitated with a mixed solvent of ethanol/acetone (1:1, v/v) and washed with acetone to obtain QCS.

2. Synthesis of quaternized chitosan maleimide butanamide (Mal-QCS)

Maleimide butyric acid (0.0366 g, 0.2 mmol), EDC (0.0198 g, 0.2 mmol) and NHS (0.023 g, 0.2 mmol) were dissolved in 0.5 mL of DMSO and stirred at room temperature for 1 h. The reaction solution was slowly dropped into QCS (162 mg) solution which pH was adjusted to 5.5 with hydrochloric acid, and stirred in an ice bath for 4 h. After reaction, the mixture was dialyzed with deionized water for 2 d and finally lyophilized to obtain Mal-QCS.

3. Synthesis of thiol terminal PBAE (SH-PBAE)

The synthesis was divided into two steps. The first step was to synthesize the PBAE with acrylate end group according to our previous work, the second step was to synthesize the sulfhydryl terminal PBAE. Previously synthesized PBAE was dissolved in 3 mL DMF, and then slowly dropped into 2 mL DMF containing 5 mmol DTT, and stirred for 2 d at room temperature. After the reaction, the solution was precipitated in ether, washed twice and dried under vacuum.

4. Synthesis of DTT-MAL

Maleimide butyric acid (0.1832 g, 1 mmol) and DTT (0.0386 g, 0.25 mmol) were dissolved in 2.2 mL of acetate buffer (pH 5.0) for 4 h in an ice bath. After reaction, the mixture was lyophilized to obtain DTT-MAL.

Table S1. TE and LCC of polymer-GFP NPs on HEK293T and ME180 cells.

			HEK293T		ME180	
	**mass ratio**	**N/P**	**TE (%)**	**LCC (%)**	**TE (%)**	**LCC (%)**
PBAE:QCP	blank	-	0.36	33.1	0.34	71.4
	0 : 1	116	3.01	38.1	13.7	67.3
	0.5 : 1	155	91.3	37.7	19.3	75.6
	1 : 1	194	97.0	29.8	29.3	56.6
	2 : 1	271	97.4	28.3	36.6	59.0
	4 : 1	427	97.9	25.7	35.0	53.8
	1 : 0	78	97.4	21.0	31.4	48.0
PEI:GFP	7.5:1	52	87.0	28.8	-	-
	45:1	313	-	-	6.84	34.4
PQ:GFP	blank	-	0.42	34.9	0.33	59.0
	30	108	85.4	28.5	17.4	26.6
	50	181	96.9	31.5	29.3	22.1
	75	271	98.0	29.8	37.4	28.5
	100	361	98.2	20.1	32.0	27.8

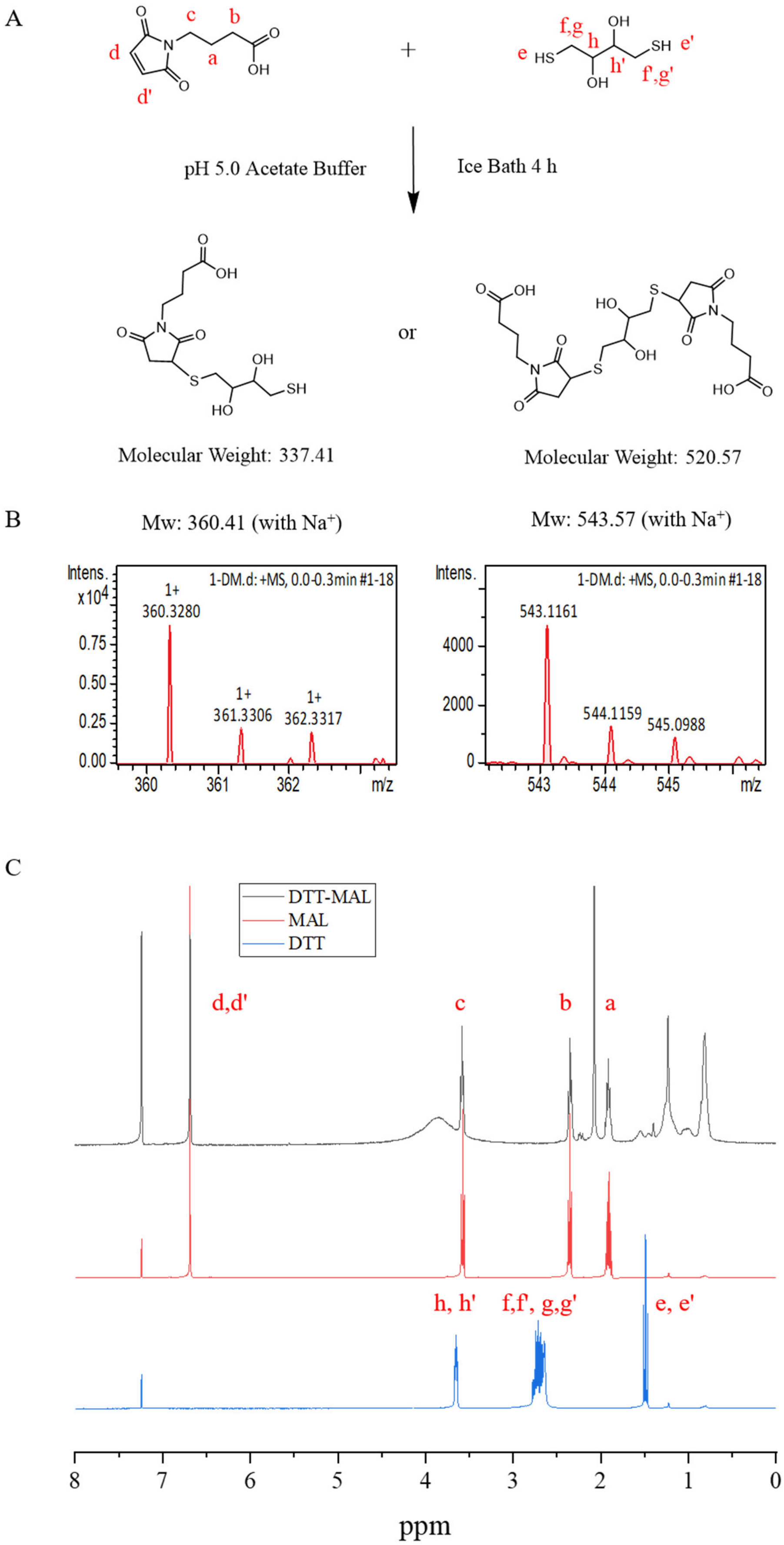

Figure S1. Synthesis and characterization of DTT-MAL. (A) Synthesis of DTT-MAL. (B) MS of DTT-MAL. (C) ^{1}H-NMR of DTT, MAL and DTT-MAL.

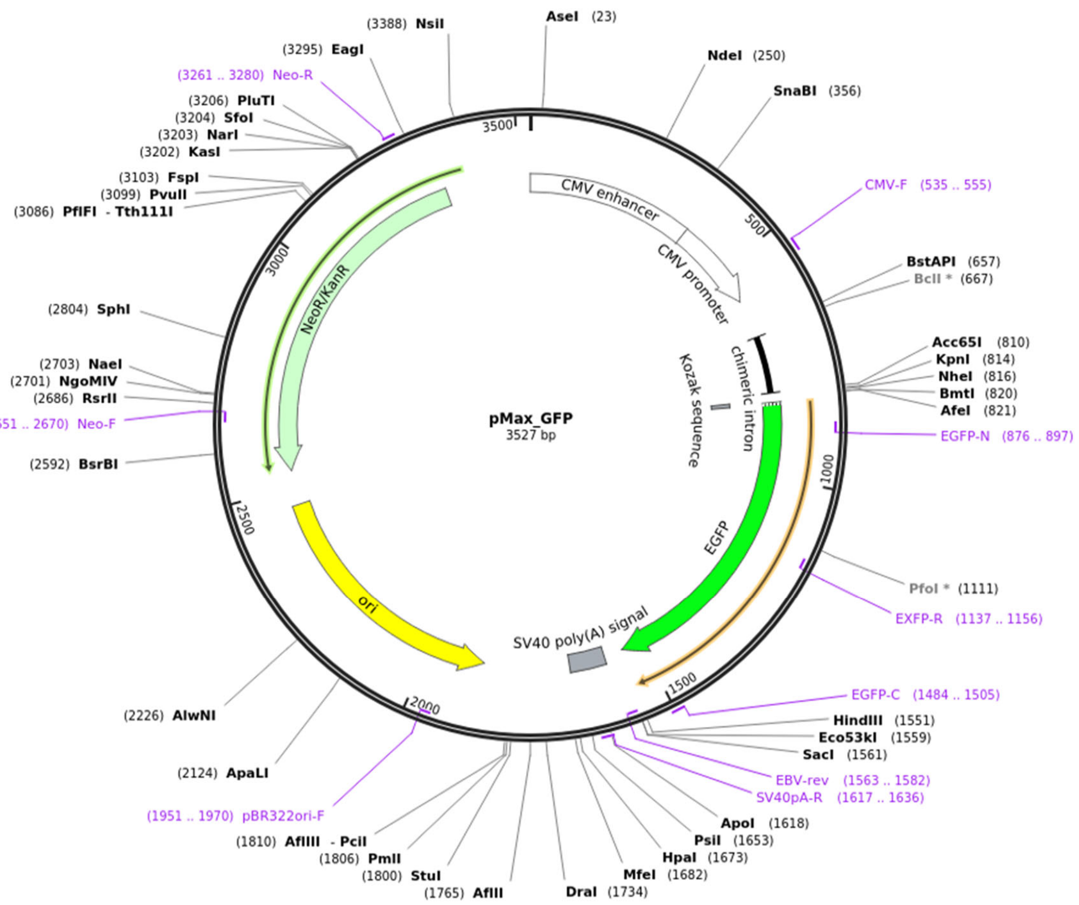

Figure S2. The vector map of pGFP.

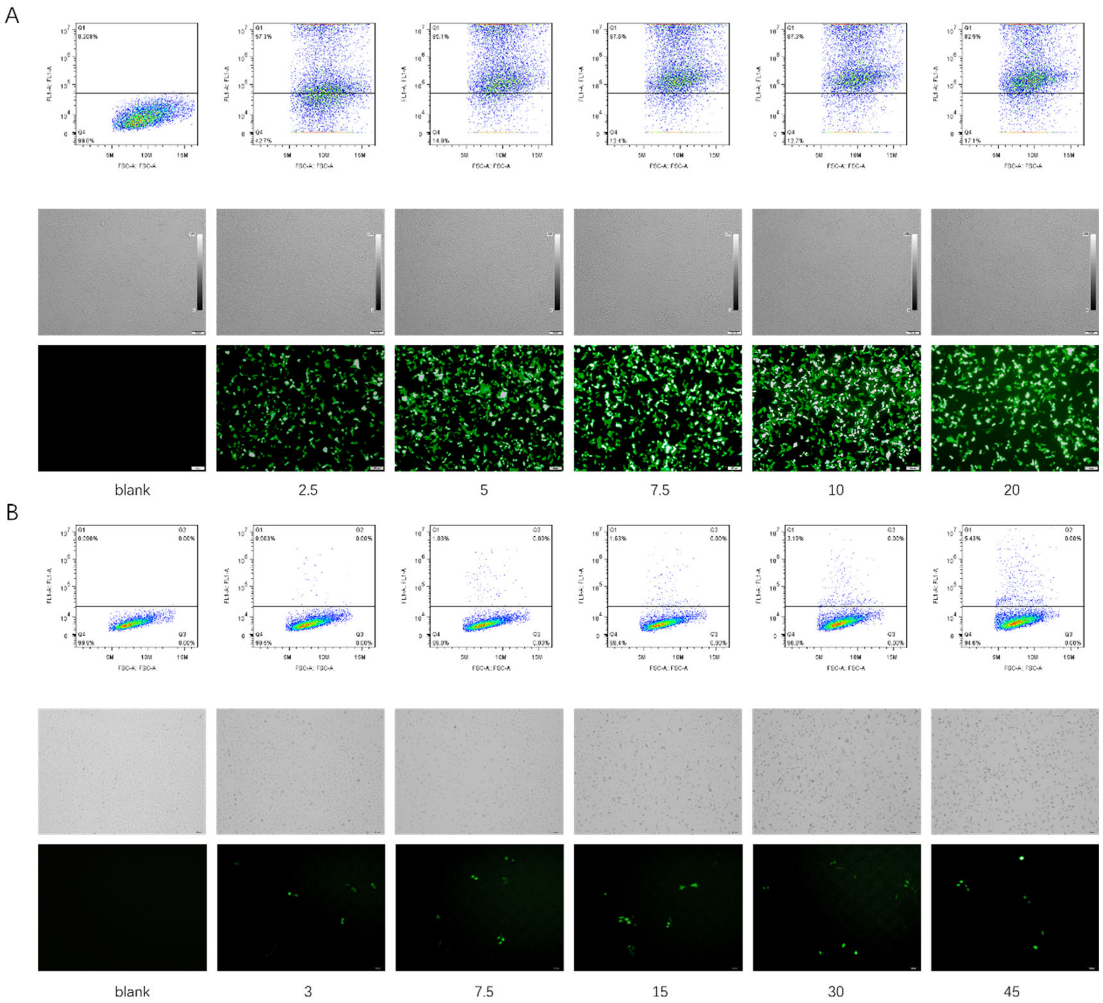

Figure S3. Flow cytometry results and fluorescence images of PEI-GFP NPs on HEK293T (A) and ME180 (B) cells at different mass ratios of PEI and GFP. Scale bar, 100 μm (A) and 50 μm (B).

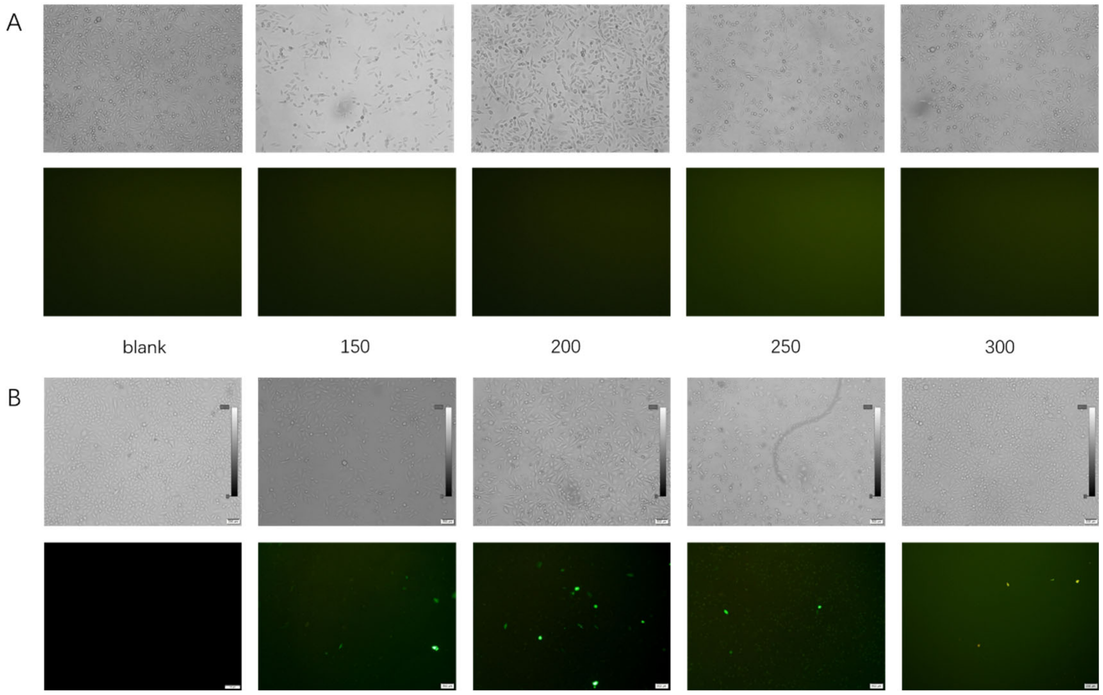

Figure S4. Transfection results of CS-GFP (A) and QCS-GFP (B) NPs in ME180 cell line. Scale bar, 200 μm.

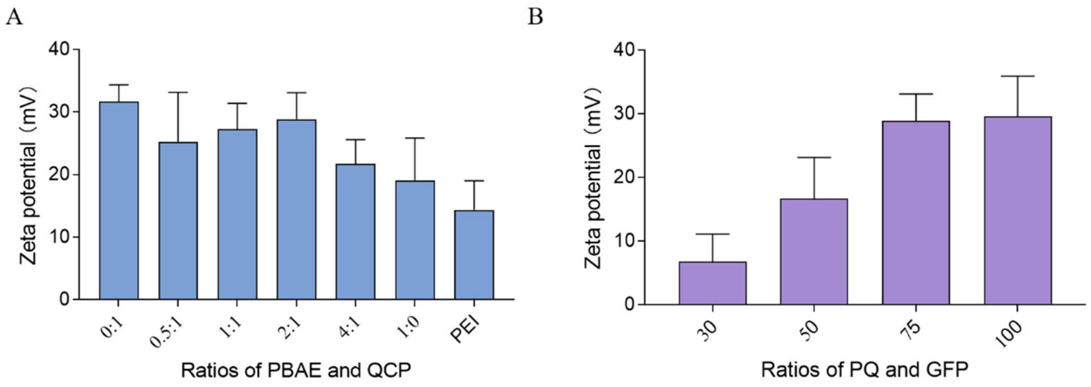

Figure S5. ζ-potential of PQ–GFP NPs at different mass ratios of PBAE and QCP (A) and mass ratios of PQ and GFP (B). Each point represents the mean ± SD (n = 3).

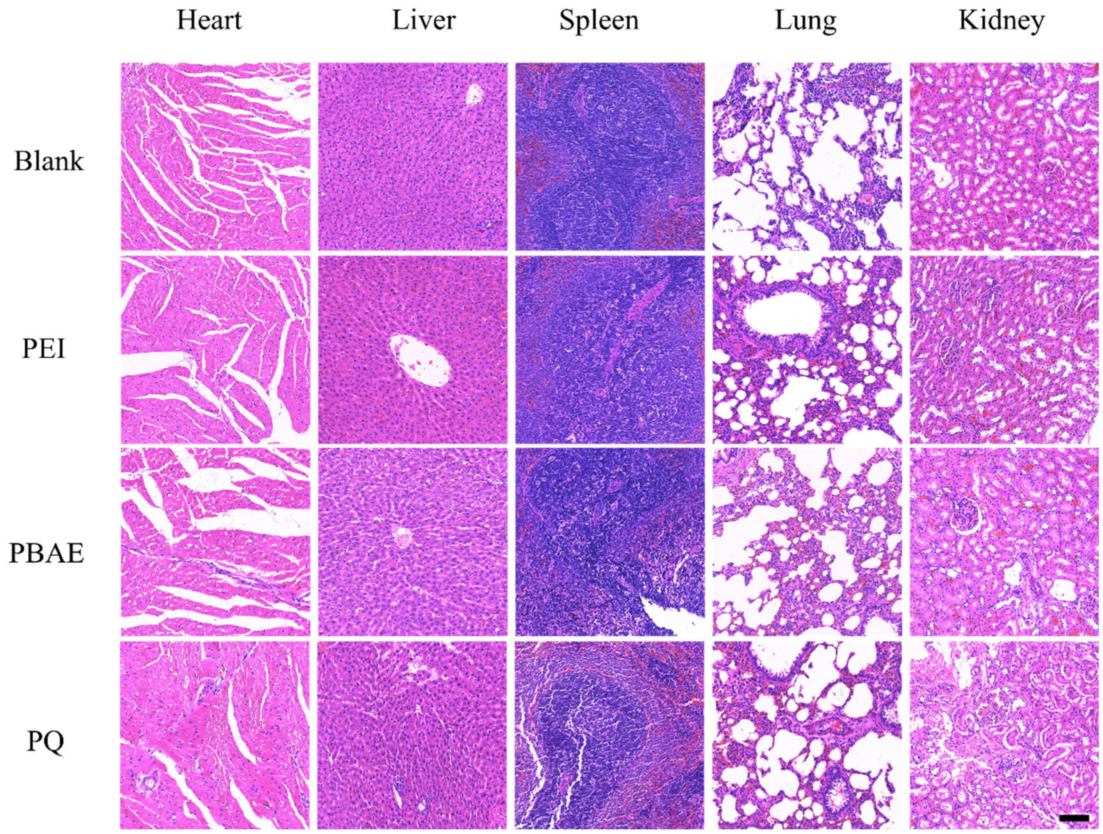

Figure S6. Representative images of H&E staining of major organs (heart, liver, spleen, lung and kidney) in SD rats treated with PEI, PBAE and PQ-GFP NPs. Scale bars, 100 µm.
